# Antileishmanial Activity and Inducible Nitric Oxide Synthase Activation by RuNO Complex

**DOI:** 10.1155/2016/2631625

**Published:** 2016-10-04

**Authors:** Tatiane Marcusso Orsini, Natalia Yoshie Kawakami, Carolina Panis, Ana Paula Fortes dos Santos Thomazelli, Fernanda Tomiotto-Pellissier, Allan Henrique Depieri Cataneo, Danielle Kian, Lucy Megumi Yamauchi, Florêncio S. Gouveia Júnior, Luiz Gonzaga de França Lopes, Rubens Cecchini, Idessânia Nazareth Costa, Jean Jerley Nogueira da Silva, Ivete Conchon-Costa, Wander Rogério Pavanelli

**Affiliations:** ^1^Department of Pathological Sciences, Center of Biological Sciences, State University of Londrina, 86057-970 Londrina, PR, Brazil; ^2^Laboratory of Inflammatory Mediators, State University of Western Paraná, 85605-010 Francisco Beltrão, PR, Brazil; ^3^Department of Microbiology, Center of Biological Sciences, State University of Londrina, 86057-970 Londrina, PR, Brazil; ^4^Department of Organic and Inorganic Chemistry, Federal University of Ceará, 60020-181 Fortaleza, CE, Brazil; ^5^Department of Chemistry, State University of Roraima, 69310-000 Boa Vista, RR, Brazil

## Abstract

Parasites of the genus* Leishmania* are capable of inhibiting effector functions of macrophages. These parasites have developed the adaptive ability to escape host defenses; for example, they inactivate the NF-*κ*B complex and suppress iNOS expression in infected macrophages, which are responsible for the production of the major antileishmanial substance nitric oxide (NO), favoring then its replication and successful infection. Metal complexes with NO have been studied as potential compounds for the treatment of certain tropical diseases, such as ruthenium compounds, known to be exogenous NO donors. In the present work, the compound *cis*-[Ru(bpy)_2_SO_3_(NO)]PF_6_, or RuNO, showed leishmanicidal activity directly and indirectly on promastigote forms of* Leishmania (Leishmania) amazonensis*. In addition, treatment with RuNO increased NO production by reversing the depletion of NO caused by* Leishmania*. We also found increased expression of Akt, iNOS, and NF-*κ*B in infected and treated macrophages. These results demonstrated that RuNO was able to kill the parasite by NO release and modulate the transcriptional capacity of the cell.

## 1. Introduction

 American cutaneous leishmaniasis (ACL) is an endemic disease in Brazil, in which the causative agents are the protozoans* Leishmania (Viannia) braziliensis *and* Leishmania (Leishmania) amazonensis*. ACL has several clinical forms: localized, disseminated, or diffuse skin lesions and aggressive and mutilating mucocutaneous wounds [1]; manifestations depend on not only the species of the parasite but also the immune response of the host [2].

 The recommended treatment of ACL is the use of pentavalent antimonials such as sodium stibogluconate (Pentostam®) and methylglucamine antimoniate (Glucantime®) [3]. However, the treatment has several side effects, such as nausea, vomiting, and hepatic and cardiotoxicity, leading patients to discontinue treatment and favoring the emergence of resistant strains [4].

*Leishmania* has several mechanisms for escaping the immune response. An important one is its ability to interfere with nitric oxide (NO) production, by suppressing the expression of inducible nitric oxide synthase (iNOS) in macrophages, which consequently results in depletion of NO, a key mediator of leishmanicidal activity, able to impair the replication of* L. amazonensis* [5].

The activation of iNOS is dependent on NF-*κ*B transcription. Parasites of the genus* Leishmania* can cleave the NF-*κ*B p65 subunit, preventing the transcription of several proinflammatory mediators and consequently affecting iNOS expression and NO production [6].

For survival,* Leishmania* can further evade cytotoxic mechanisms by reducing NO production through the increased expression of arginase, which cleaves L-arginine, a precursor of NO synthesis [7, 8].

Altogether, the lack of effective drugs and the serious side effects of those available, coupled with the inability of the host's body to deal with the parasite, have been the driving force behind the search for new chemotherapeutic agents against* Leishmania*. Therefore, NO-based complexes seem to be a promising approach.

Accordingly, the pivotal role of NO in ACL has increased interest in understanding its interaction with metal complexes, such as with ruthenium, aiming to use these compounds as possible NO donor drugs in biological conditions [9]. This kind of complex has been previously tested against* Leishmania* and shown to inhibit mitochondrial respiration in amastigote and promastigote forms, killing the parasite [10, 11].

Some complexes such as trans-[Ru(NO)(NH_3_)_4_L](X)_3_, [Ru(NO)Hedta], and Na_2_[Fe(CN)_5_NO]·H_2_O_2_ were very effective when tested against* Leishmania* spp. forms [5, 12, 13] and other pathogens such as* Trypanosoma cruzi* [14] and* Paracoccidioides brasiliensis* [15].

Ruthenium NO donors have shown desirable properties for therapeutic use, such as low cytotoxicity and high water solubility [16], and controlled release of NO [10]. Accordingly, we performed* in vitro* assays to investigate the direct effect of the complex* cis*-[Ru(NO)(bpy)_2_(SO_3_)](PF_6_) on parasites as well as its modulatory action on* Leishmania*-infected macrophages.

## 2. Materials and Methods

### 2.1. *Leishmania (Leishmania) amazonensis* Culture

Promastigote forms of* Leishmania* (*Leishmania*)* amazonensis* (MHOM/BR/1989/166MJO) were maintained in 199 media (GIBCO Invitrogen), supplemented with 10% fetal bovine serum (FBS, GIBCO Invitrogen), 1 M Hepes (4-(2-hydroxyethyl)-1-piperazine ethanesulfonic acid), 0.1% human urine, 0.1% L-glutamine, 10 U/mL penicillin and 10 *μ*g/mL streptomycin (GIBCO Invitrogen), and 10% sodium bicarbonate. Promastigote cultures were incubated at 24°C in 25-cm^2^ flasks for 5 days.

### 2.2. Nitric Oxide Donor

Nitric oxide donor RuNO complex was synthesized and characterized by researchers [17] in the Department of Organic and Inorganic Chemical, University of Ceará-UFC.

### 2.3. Antiprotozoal Activity

Promastigote forms (1 × 10^6^/mL) grown in 199 media were incubated with 30 and 60 *μ*M RuNO complex at 24°C and were counted in a Neubauer chamber after 24 h. Promastigotes grown in the culture medium alone served as the control.

### 2.4. Viability of Peritoneal Macrophages

Macrophages (3 × 10^6^/mL) were obtained from the peritoneal cavity of BALB/c mice, resuspended in RPMI 1640 culture medium (GIBCO) supplemented with 10% FBS, and distributed in 24-well plates for 2 h of adherence in 5% CO_2_ at 37°C. After adherence, cells were treated with different concentrations of RuNO complex (10–640 *μ*M); the positive control contained only RPMI 1640 and the negative control contained 4% H_2_O_2_. After 24 h of treatment, MTT (3-(4,5-dimethylthiazol-2-yl)-2,5-diphenyltetrazolium bromide) was added, and the plate was incubated for 4 h. The MTT-formazan crystals that formed were dissolved in DMSO (dimethylsulfoxide) and the absorbance was measured at 540 nm using a spectrophotometer [18].

### 2.5. Phagocytic Assay

Peritoneal macrophages (5 × 10^5^/mL) were obtained by injection of 3 mL of RPMI 1640 culture medium (GIBCO®) supplemented with 10% FBS (GIBCO) and grown in 24-well plates containing 13-mm diameter glass coverslips. Cells were preincubated with 200 *μ*L of RPMI medium for 2 h for adherence, and, afterwards, the cells were washed with saline to remove nonadherent cells. Adherent cells were then infected with promastigote forms (1 : 5) for 2 h, treated with RuNO complex (30 and 60 *μ*M) or with RPMI medium alone (control), and incubated for 24 h at 37°C/5% CO_2_. Cells were stained with Giemsa to determine the number of infected macrophages and parasites/macrophage.

### 2.6. Promastigote Recovery

Peritoneal macrophages (5 × 10^5^/mL) were incubated in 24-well plates, and, after 2 h, macrophages were infected with promastigotes (1 : 5) for 2 h. The culture was washed to remove extracellular parasites and incubated with 199 culture media at 24°C, and the cells were treated with 60 *μ*M RuNO complex plus L-NAME (NG-nitro-L-arginine methyl ester). Recovered promastigotes were counted in a Neubauer chamber for four days.

### 2.7. Cytokine Determination

Supernatants in the phagocytic assay were collected, centrifuged at 460 ×g at 4°C for 7 min, and stored at 20°C for cytokine determination. TNF-*α* (tumor necrosis factor alpha), IL- (interleukin-) 1*β*, IL-10, IFN-*γ*, TGF-*β*, and IL-12 were determined by enzyme-linked immunosorbent assay (ELISA), according to the manufacturer's instructions (eBiosciences®, USA). Plates were read at 450 nm, using an ELISA plate reader (Thermo Plate—TP-Reader).

### 2.8. Determination of Nitrite Levels

Nitrite was measured in the supernatant of macrophages infected and then treated with nitric oxide donor [19]. Briefly, supernatants in phagocytic assays were deproteinized by adding zinc sulfate (ZnSO_4_) solution and sodium hydroxide (NaOH). The mixture was centrifuged (10,845 ×g, 5 min, 4°C). The supernatant was recovered and diluted in glycine buffer. Cadmium granules were rinsed in sterile distilled water and added to a copper sulfate (CuSO_4_) solution, which was allowed to stand for 5 min, and the copper-coated cadmium granules were then used within 10 min. Activated granules were added to glycine buffer-diluted supernatant and stirred for 10 min. Aliquots of 50 *μ*L of the supernatant aliquots were transferred to 96-well microplates and an equal volume of Griess reagent was added. A calibration curve was prepared using sodium nitrite (NaNO_2_) at different concentrations, and the absorbance was determined at 550 nm in a microplate reader.

### 2.9. NOS Inhibition Assay

Peritoneal macrophages were stimulated with lipopolysaccharide (LPS), and others were subjected to infection and treatment with 60 *μ*M RuNO. However, before treatment with RuNO complex, the cells were incubated with 100 *μ*M L-NAME at 36°C and 5% CO_2_ for 24 h. The culture supernatant was used to measure NO levels (by determination of nitrite levels as previously described).

### 2.10. Immunocytochemistry for Akt, NF-*κ*B, and iNOS

Immunocytochemistry (ICC) for Akt, NF-*κ*B (p65), and iNOS was performed on coverslip-adherent cells (cells prepared as in the protocol described for the phagocytic assay) using the labeled streptavidin biotin method with the LSAB kit (DAKO Japan, Kyoto, Japan). The coverslips were incubated with Triton X-100 solution for 1 h, washed in PBS, and treated at room temperature with 10% BSA. Next, coverslips were incubated overnight at 4°C with mouse primary antibody (anti-Akt, anti-NF-*κ*B, and anti-iNOS rabbit polyclonal antibody diluted 1 : 200; Santa Cruz Biotechnology, USA). After secondary antibody treatment (2 h, room temperature), horseradish peroxidase activity was visualized by treatment with H_2_O_2_ and 3,3′-diaminobenzidine (DAB) for 5 min. In the last step, the coverslips were weakly counterstained with Harry's hematoxylin (Merck). For each case, negative controls were performed by omitting the primary antibody. Intensity and localization of immunoreactivity against the primary antibody used were examined on all coverslips using a photomicroscope (Olympus BX41, Olympus Optical Co., Ltd., Tokyo, Japan). For the image analysis study, photomicroscopic color slides of representative areas (40x objective lens) were digitally acquired. For determining a semiquantitative scoring, images were evaluated by using the color deconvolution tool from the Image J software (NIH, USA). Pixels were categorized as previously described by [20] as high positive (3+), positive (2+), low positive (1+), and negative (0). For nuclear staining, anti-NF-*κ*B p65 phospho S536 antibody (Abcam, catalog number ab86299, diluted 1 : 300) and anti-Akt1 phospho S473 antibody (EP2109Y Abcam, catalog number ab81283, diluted 1 : 250) were used. Coverslips were processed as described, and the quantification of nuclear staining was performed by determining the percentage of labeled cells in 30 fields/experimental conditions, in triplicate.

### 2.11. Relative Quantification of iNOS mRNA by Real Time Reverse Transcription-Polymerase Chain Reaction (RT-PCR)

RNA extraction was performed with 10^6^ cells using SV Total RNA Isolation System (Promega, USA) following the manufacturer's procedure. RNA concentration was determined by absorbance (260 nm) measurements with a spectrophotometer (Synergy HT, Biotek, USA). Complementary DNA was synthesized using 500 ng of total RNA in a reverse transcription reaction by MMLV reverse transcriptase (Invitrogen, USA) following the manufacturer's procedure. Real time RT-PCR quantitative mRNA analyses were performed in Rotor-Gene Q equipment (Qiagen, Germany) using Platinum® SYBR® Green qPCR SuperMix-UDG (Invitrogen, USA), in a final volume of 20 *μ*L. The reaction mixture also contained 2 *μ*M primers and 100 ng of cDNA template. The sequences of primers used for inducible nitric oxide synthase were iNOS-F 5′-cgaaacgcttcacttccaa-3′ and iNOS-R 5′-tgagcctatattgctgtggct-3′ and for *β*-actin were b-actin-F 5′-agctgcgttttacacccttt-3′ and b-actin-R 5′-aagccatgccaatgttgtct-3′. Cycling conditions were 10 min at 95°C and 40 cycles of 30 s at 95°C, 30 s at 62°C, and 30 s at 72°C, followed by melting curve analysis (60 to 95°C at 0.5°C/s). Gene expression levels were determined with reference to *β*-actin using the comparative cycle threshold method.

### 2.12. Statistical Analysis

Experiments were performed in triplicate. Data were obtained from three independent experiments and expressed as mean ± standard error of the mean. Statistical analyses were conducted by using the GraphPad Prism software (version 5.0). Groups were compared by using one-way ANOVA and Tukey's test. *P* < 0.05 was considered significant.

## 3. Results

### 3.1. Nitric Oxide Donor* cis*-[Ru(NO)(bpy)_2_(SO_3_)](PF_6_) Exhibits Antileishmanial Effect

**Figure 1:**
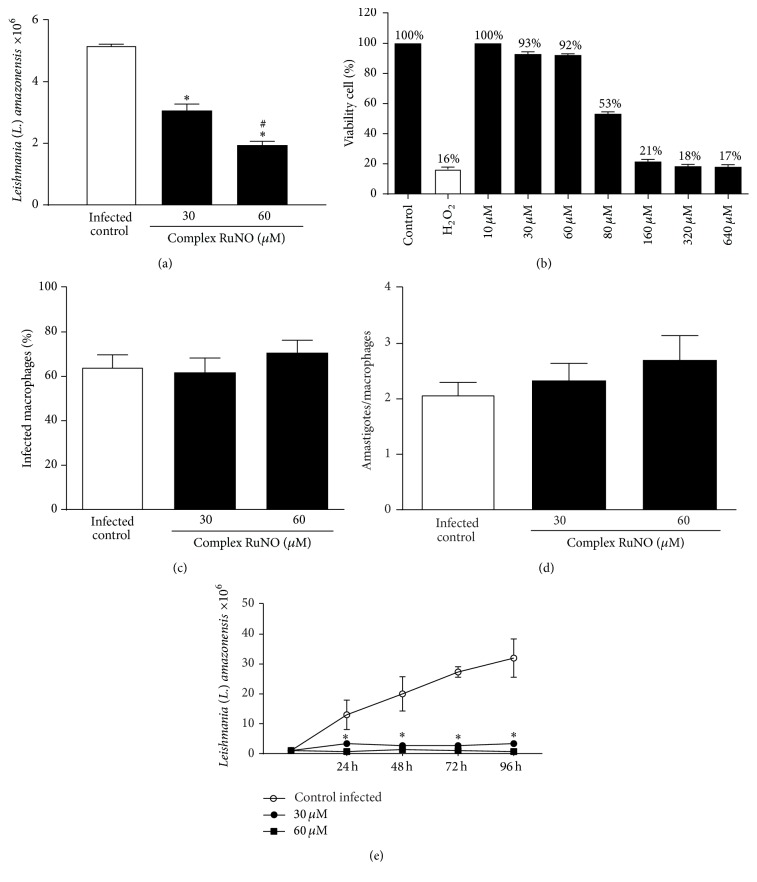
Antileishmanial activity by ruthenium complex. (a) Amount of* L. amazonensis* promastigote forms after treatment with RuNO complex (30 and 60 *μ*M) for 24 h. [Values represent the mean ± SEM of three independent experiments (*P* < 0.05). ^*∗*^Significantly different from infected control. ^#^Significantly different between concentrations]. (b) Viability of peritoneal macrophages of BALB/c mice treated with RuNO complex (10–640 *μ*M) for 24 h. [Values represent the mean ± SEM of three independent experiments]. (c) Percentage of infected macrophages after 24 h of incubation with RuNO complex (30 and 60 *μ*M). (d) Mean number of amastigotes per macrophage after 24 h of incubation with RuNO complex (30 and 60 *μ*M). (e) Recovery kinetics of promastigotes that had infected peritoneal macrophages, which were then treated with RuNO complex (30 and 60 *μ*M). [Values represent the mean ± SEM of three independent experiments (*P* < 0.05). ^*∗*^Significantly different from control.]

The antileishmanial effect of RuNO complex was evaluated by determining the proliferation of promastigote forms of* L. amazonensis*. Parasites were incubated with RuNO complex at two different concentrations (30 and 60 *μ*M) for 24 h. We observed that RuNO at the concentrations used significantly inhibited the proliferation of* L. amazonensis* when compared with control group, demonstrating that RuNO complex had antileishmanial activity (Figure 1(a), *P* < 0.05).

According to MTT cell viability assays, we found that RuNO complex was not toxic at 10 to 60 *μ*M (Figure 1(b)).

### 3.2. Nitric Oxide Donor Does Not Change Phagocytic Capacity but Affects the Release of Promastigote Forms from Macrophages

To see if treatment with RuNO complex could modify the phagocytic capacity of macrophages, we incubated these cells with 30 and 60 *μ*M RuNO for 24 h and then evaluated the phagocytic index by determining the mean number of amastigotes per macrophage. We found that the treatment did not alter the percentage of infected macrophages as well as the number of amastigotes per macrophage (Figures 1(c) and 1(d), *P* < 0.05). However, when we determined the recovery of promastigotes, we observed that all concentrations significantly reduced the number of parasites recovered when compared to the control group (Figure 1(e), *P* < 0.05).

### 3.3. RuNO Complex Does Not Modulate Cytokine Pattern in Infected Macrophages

**Figure 2:**
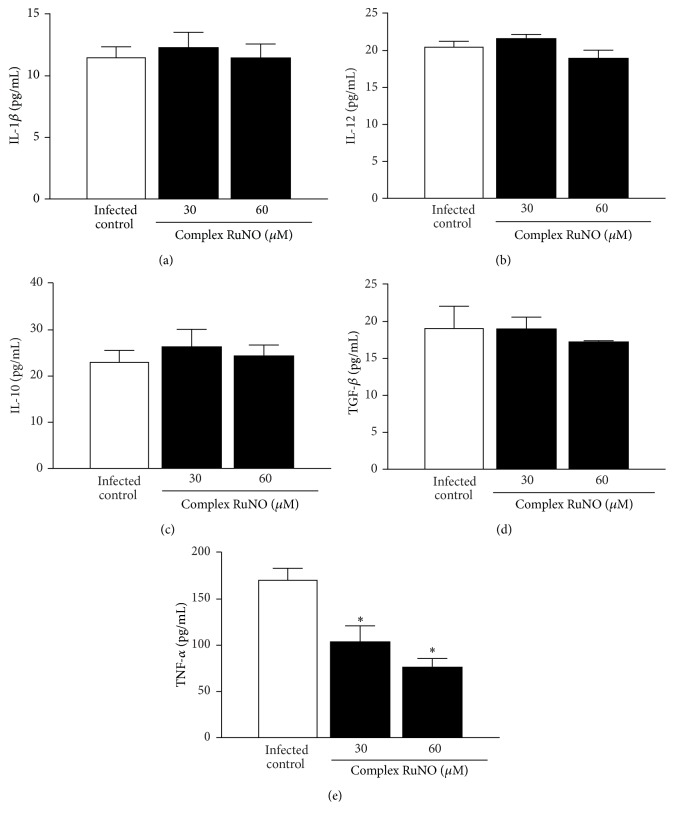
*In vitro* production of cytokines by peritoneal macrophages infected and then treated with RuNO complex (30 and 60 *μ*M). (a) IL-1*β*, (b) IL-12, (c) IL-10, (d) TGF-*β*, and (e) TNF-*α*. [Values represent the mean ± SEM of three independent experiments (*P* < 0.05). ^*∗*^Significantly different from control.]

Aiming to understand the mechanism by which RuNO complex kills the parasite, we evaluated the cytokine profile of macrophages during* Leishmania* infection. After infection of macrophages, only TNF-*α* production was significantly decreased when compared with control (Figure 2, *P* < 0.05).

### 3.4. RuNO Complex Increases Nitric Oxide Levels

**Figure 3:**
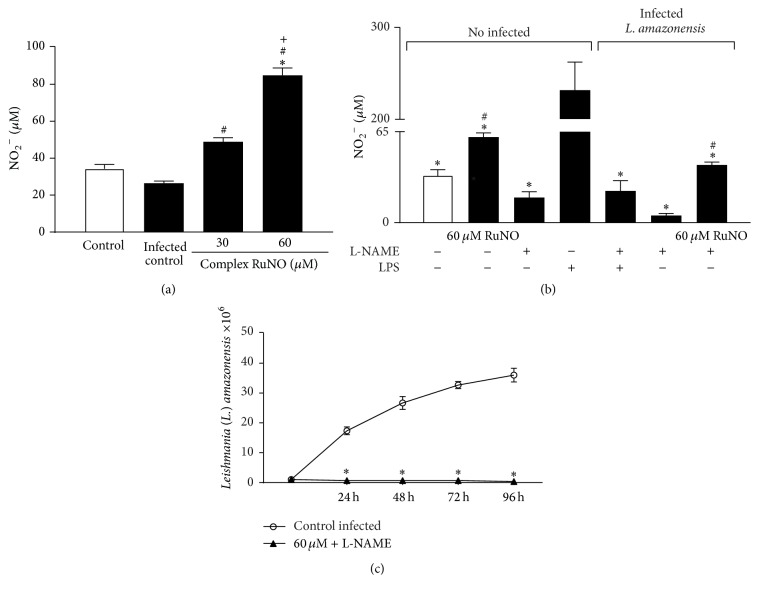
(a) Nitrite levels of macrophages infected and then treated with RuNO complex (30 and 60 *μ*M) for 24 h [^*∗*^significantly different from control, ^#^significantly different from infected control, and ^+^significantly different from concentrations]. (b) Determination of nitrite levels of peritoneal macrophages stimulated with LPS, infected with* L. amazonensis*, and then treated with 100 *μ*M L-NAME and 60 *μ*M RuNO complex for 24 h. [Values represent the mean ± SEM of three independent experiments (*P* < 0.05). ^*∗*^significantly different from control LPS and ^#^significantly different from infected control with L-NAME]. (c) Recovery kinetics of promastigotes in peritoneal macrophages infected with* L. amazonensis*, treated with RuNO complex (60 *μ*M) and the iNOS inhibitor L-NAME (100 *μ*M) for 24 h. [^*∗*^significantly different from infected control at all times.]

Regarding NO levels, the results revealed that treatment with RuNO complex significantly increased nitrite levels in infected macrophages (Figure 3(a), *P* < 0.05). To determine whether NO found in the macrophage culture supernatant was endogenous or exogenous, we blocked NOS activity with L-NAME. We observed that treatment with RuNO complex plus L-NAME induced an increase in nitrite levels (Figure 3(b), *P* < 0.05). Additionally, the treatment caused a significant reduction in the amount of promastigotes recovered (Figure 3(c), *P* < 0.05). Together, these results suggested that NO was responsible for the death of parasites and came from the NO donor.

### 3.5. RuNO Complex Increases the Expression of Akt, iNOS, and NF-*κ*B (p65) in Macrophages

**Figure 4:**
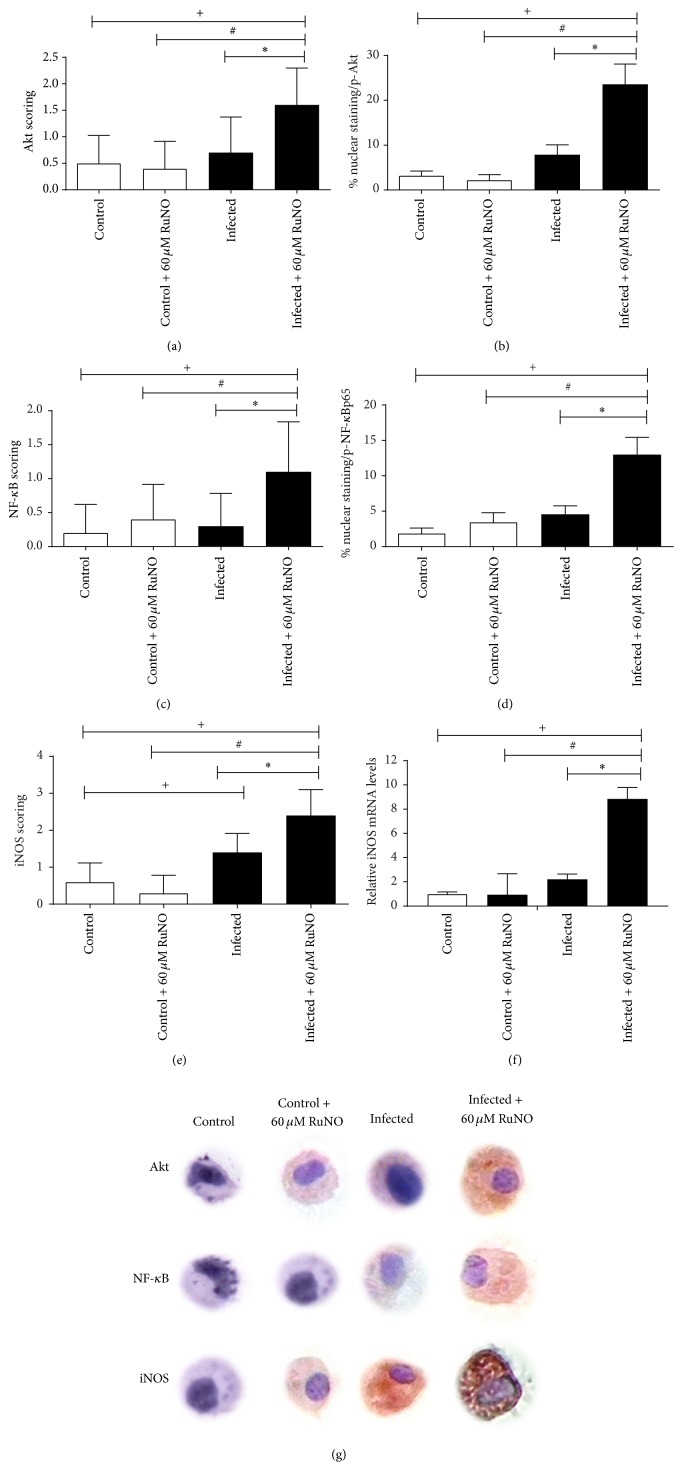
ICC scoring, nuclear staining, and relative quantification of iNOS mRNA of macrophages infected with* L. amazonensis* and treated with RuNO complex (60 *μ*M) for 24 h. (a) Akt, (b) phosphorylated Akt (P-Akt), (c) NF-*κ*B, (d) phosphorylated p65 (P-p65), (e) iNOS, (f) iNOS mRNA, and (g) immunocytochemical staining. [Values represent the mean ± SEM of three independent experiments (*P* < 0.05). [^*∗*^significantly different from control infected, ^#^significantly different from treated control, and ^+^significantly different from control.]

ICC results showed a significant increase in Akt, NF-*κ*B, and iNOS labeling in infected macrophages treated with RuNO complex (Figures 4(a), 4(c), and 4(e), *P* < 0.05). To confirm these results, we analyzed the percentage of phosphorylated p65 (P-p65) and phosphorylated Akt (P-Akt). The results showed a significant increase in phosphorylated nuclear Akt and p65 in infected macrophages treated with RuNO complex (Figures 4(b) and 4(d), *P* < 0.05). Similarly, to better understand the mechanism underlying the increase in iNOS labeling by RuNO, we analyzed its effect on iNOS mRNA expression. The results revealed that treatment significantly increased iNOS mRNA expression in infected macrophages (Figure 4(e), *P* < 0.05).

## 4. Discussion

It is well known that NO is one of the crucial molecules in the control of parasite load during the development of ACL [21]. To evade host immunity, parasites of the genus* Leishmania* modulate the response in macrophages by decreasing iNOS activity and NO production by depleting the enzyme substrate, as well as increasing the production of essential polyamines that are needed for the growth and differentiation of the parasites [8]. So far, there is still no known drug capable of restoring NO levels in macrophages.

In this study, we demonstrated* in vitro* that the NO donor RuNO complex showed antileishmanial activity by releasing NO and eliminating promastigote forms of* L. amazonensis*. Our group has previously demonstrated [14, 22] that NO donors based on ruthenium complexes display potent trypanocidal activity. Another study also demonstrated such antileishmanial activity but with another compound and with* L. major* [13].

In the present study, we demonstrated that the treatment with RuNO complex did not interfere with the phagocytic activity of macrophages; however, macrophages were able to kill the parasite. Another study [23] reported that when high concentrations of NO were produced* in vitro*, axenic amastigotes of* L. amazonensis* were susceptible. Another NO donor showed trypanocidal activity* in vitro* in Vero cells treated after infection with* T. cruzi*, which demonstrated the effectiveness of the treatment based on the use of exogenous NO donors [24]. The authors also concluded that the NO donor, utilized both* in vitro* and* in vivo*, had trypanocidal activity and was capable of inducing a parasitological cure in a mouse model.

Beyond NO, experimental models have shown that the outcome of* Leishmania* infection is critically dependent on the immune response. In fact, the susceptibility to this disease is ascribed to a Th2 immune response, which leads to the development and multiplication of the parasite [25]. On the other hand, resistance is established by preferential activation of the Th1 subpopulation of lymphocytes, which leads to the production of many cytokines, particularly IFN-*γ* and TNF-*α*; these cytokines can activate macrophages, increasing iNOS activity, which in turn increases the production of nitric oxide [26, 27].

Despite the immunomodulation that RuNO complex induced in peritoneal macrophages, the parasites can modulate the whole immune response. Gomes et al. [28] also reported that macrophages infected with* L. amazonensis* produced low levels of TNF-*α* when compared to macrophages infected with* L. major*. This explains the low production of TNF-*α*.

Kima (2007) [29] established that promastigotes and amastigotes of all species are capable of inhibiting the production of IL-12 from active cells. It is known that two stimuli are required for the expression and activation of iNOS; the first stimulus is IFN-*γ*, which is followed by a second signal mediated by TNF-*α*, resulting in sustained NO production [23].

In our work, there were no high levels of these two cytokines, but when analyzed by ICC and RT-PCR, iNOS expression was found to be increased, suggesting that the RuNO complex can directly induce the enzyme, thereby increasing NO production.

In the present work, RuNO complex was able to increase the induction of Akt and consequently induce iNOS and NF-*κ*B activation (Figure 4). It is possible that Akt acts in I*κ*B degradation and translocation of NF-*κ*B to the nucleus, promoting iNOS production.

Some studies have reported the importance of the Akt pathway in epithelial cells and macrophages. It has been suggested that this pathway plays a significant role in signal transduction involved in the induction of iNOS and NF-*κ*B activation, stimulating the activation and release of NF-*κ*B through the degradation of I*κ*B [30–32].

Some studies have reported that NO positively regulates iNOS expression in mouse cell types and in human cells [33, 34]. Lee and Choy [35] reported that the inhibition of iNOS activity attenuated the expression of iNOS protein and that the addition of exogenous nitric oxide donor was able to restore iNOS levels. The increase in NO by iNOS expression was due to Ras nitrosylation, which increased levels of iNOS by sustained Akt activation.

We demonstrated that treatment with RuNO complex increased NO levels and that the majority of NO measured came from the donor, confirming the ability of RuNO complex to release NO.

In fact, by blocking iNOS with L-NAME, we proved that most of the NO detected (Figure 3(c)) was from RuNO complex and that this was responsible for the death of the parasites.

Genestra et al. [36] demonstrated that peritoneal macrophages from BALB/c mice infected with* L. amazonensis* showed elevated NO levels after 24 h. Furthermore, when macrophages were incubated with L-NAME for 24 h, NO production was significantly reduced. Blocking NO synthesis with L-NAME was clearly seen, which was not reversed by the addition of L-arginine, and peritoneal macrophages treated with L-NAME showed a significantly lower infection index after 24 h. This report indicated that the NO pathway is truly important in the establishment of infection. Perrella Balestieri et al. [37] suggested that the increase in the number of parasites in phagolysosomes leads to decreased production of NO by iNOS.

Therefore, the use of compounds that provide for the delivery of NO molecules, perhaps the best compounds with leishmanicidal activity, can be a viable alternative for the treatment of leishmaniasis. Accordingly, the NO donor used in the present study may be one of these possibilities, because it was able to kill the parasite through the release of NO, as well as modulating the transcriptional capacity of the phagocytic cells.
